# Supplementary material for: Inversion modeling of japonica rice canopy chlorophyll content with UAV hyperspectral remote sensing
Source: PLoS One. 2020 Sep 11;15(9):e0238530. doi: 10.1371/journal.pone.0238530 (PMC7485794; doi:10.1371/journal.pone.0238530)

PLOS ONE

Inversion Modeling of Japonica Rice Canopy

Chlorophyll Content with UAV Hyperspectral

Remote Sensing

--Manuscript Draft--

**Manuscript Number:**	PONE-D-20-07987
**Article Type:**	Research Article
**Full Title:**	Inversion Modeling of Japonica Rice Canopy<br><br>Chlorophyll Content with UAV Hyperspectral<br><br>Remote Sensing
**Short Title:**	Inversion Modeling of Japonica Rice Canopy<br>Chlorophyll Content
**Corresponding Author:**	Xu Tongyu<br>Shenyang Agricultural University<br>Shenyang, CHINA
**Keywords:**	
**Abstract:**	Abstract—Chlorophyll content is an important indicator of the growth status of japonica rice. The objective of this paper is to develop an inversion model that can predict japonica rice chlorophyll content by using hyperspectral image of rice canopy collected with unmanned aerial vehicle (UAV). UAV-based hyperspectral remote sensing can provide timely and cost-effective monitoring of chlorophyll content over a large region. The study was based on hyperspectral data collected at the Shenyang Agricultural College Academician Japonica Rice Experimental Base in 2018 and 2019. In order to extract the salient information embedded in the high-dimensional hyperspectral data, we first perform dimension reduction by using a successive projection algorithm (SPA). The SPA extracts the characteristic hyperspectral bands that are used as input to the inversion model. The characteristic bands extracted by SPA are 410 nm, 481 nm, 533 nm, 702 nm, and 798 nm, respectively. The inversion model is developed by using an extreme learning machine (ELM), the parameters of which are optimized by using particle swarm optimization (PSO). The PSO-ELM algorithm can accurately

Powered by Editorial Manager® and ProduXion Manager® from Aries Systems Corporation

model the nonlinear relationship between hyperspectral data and chlorophyll content. The model achieves a coefficient of determination R 2 = 0.791 and a root mean square error of RMSE = 8.215. The model exhibits good predictive ability and can provide data support and model reference for research on nutrient diagnosis of japonica rice.

Index Terms—UAV remote sensing; chlorophyll; japonica rice; hyperspectral; extreme learning machine; particle swarm algorithm; successive projection algorithm.

Order of Authors:	Yingli Cao
	Kailun Jiang
	Jingxian Wu
	Fenghua Yu
	Wen Du
	Xu Tongyu

Additional Information:	
**Question**	**Response**
**Financial Disclosure**<br><br>Enter a financial disclosure statement that describes the sources of funding for the work included in this submission. Review the submission guidelines for detailed requirements. View published research articles from *PLOS ONE* for specific examples.<br><br>This statement is required for submission and **will appear in the published article** if the submission is accepted. Please make sure it is accurate.	The work was supported by National Key R&D Program of China (2016YFD0200700)

Powered by Editorial Manager® and ProduXion Manager® from Aries Systems Corporation

**Unfunded studies**<br>Enter: *The author(s) received no specific funding for this work.*<br><br>**Funded studies**<br>Enter a statement with the following details:<br>• Initials of the authors who received each award<br>• Grant numbers awarded to each author<br>• The full name of each funder<br>• URL of each funder website<br>• Did the sponsors or funders play any role in the study design, data collection and analysis, decision to publish, or preparation of the manuscript?<br>• **NO** - Include this sentence at the end of your statement: *The funders had no role in study design, data collection and analysis, decision to publish, or preparation of the manuscript.*<br>• **YES** - Specify the role(s) played.<br><br>* typeset	
**Competing Interests**<br><br>Use the instructions below to enter a competing interest statement for this submission. On behalf of all authors, disclose any competing interests that could be perceived to bias this work—acknowledging all financial support and any other relevant financial or non-financial competing interests.<br><br>This statement **will appear in the published article** if the submission is accepted. Please make sure it is accurate. View published research articles from *PLOS ONE* for specific examples.	The authors have declared that no competing interests exist.

**NO authors have competing interests**<br><br>Enter: *The authors have declared that no competing interests exist.*<br><br>**Authors with competing interests**<br><br>Enter competing interest details beginning with this statement:<br><br>*I have read the journal's policy and the authors of this manuscript have the following competing interests: [insert competing interests here]*<br><br>* typeset	
**Ethics Statement**<br><br>Enter an ethics statement for this submission. This statement is required if the study involved:<br><br>• Human participants<br>• Human specimens or tissue<br>• Vertebrate animals or cephalopods<br>• Vertebrate embryos or tissues<br>• Field research<br><br>Write "N/A" if the submission does not require an ethics statement.<br><br>General guidance is provided below. Consult the submission guidelines for detailed instructions. **Make sure that all information entered here is included in the Methods section of the manuscript.**	N/A

Powered by Editorial Manager® and ProduXion Manager® from Aries Systems Corporation

**Format for specific study types**<br><br>**Human Subject Research (involving human participants and/or tissue)**<br>• Give the name of the institutional review board or ethics committee that approved the study<br>• Include the approval number and/or a statement indicating approval of this research<br>• Indicate the form of consent obtained (written/oral) or the reason that consent was not obtained (e.g. the data were analyzed anonymously)<br><br>**Animal Research (involving vertebrate animals, embryos or tissues)**<br>• Provide the name of the Institutional Animal Care and Use Committee (IACUC) or other relevant ethics board that reviewed the study protocol, and indicate whether they approved this research or granted a formal waiver of ethical approval<br>• Include an approval number if one was obtained<br>• If the study involved *non-human primates*, add *additional details* about animal welfare and steps taken to ameliorate suffering<br>• If anesthesia, euthanasia, or any kind of animal sacrifice is part of the study, include briefly which substances and/or methods were applied<br><br>**Field Research**<br><br>Include the following details if this study involves the collection of plant, animal, or other materials from a natural setting:<br>• Field permit number<br>• Name of the institution or relevant body that granted permission	
**Data Availability**<br><br>Authors are required to make all data underlying the findings described fully available, without restriction, and from the time of publication. PLOS allows rare exceptions to address legal and ethical concerns. See the PLOS Data Policy and FAQ for detailed information.	Yes - all data are fully available without restriction

A Data Availability Statement describing where the data can be found is required at submission. Your answers to this question constitute the Data Availability Statement and **will be published in the article**, if accepted.<br><br>**Important:** Stating 'data available on request from the author' is not sufficient. If your data are only available upon request, select 'No' for the first question and explain your exceptional situation in the text box.<br><br>Do the authors confirm that all data underlying the findings described in their manuscript are fully available without restriction?	
**Describe where the data may be found in full sentences. If you are copying our sample text, replace any instances of XXX with the appropriate details.**<br><br>• If the data are **held or will be held in a public repository**, include URLs, accession numbers or DOIs. If this information will only be available after acceptance, indicate this by ticking the box below. For example: *All XXX files are available from the XXX database (accession number(s) XXX, XXX.).*<br>• If the data are all contained **within the manuscript and/or Supporting Information files**, enter the following: *All relevant data are within the manuscript and its Supporting Information files.*<br>• If neither of these applies but you are able to provide **details of access elsewhere**, with or without limitations, please do so. For example:<br><br>*Data cannot be shared publicly because of [XXX]. Data are available from the XXX Institutional Data Access / Ethics Committee (contact via XXX) for researchers who meet the criteria for access to confidential data.*<br><br>*The data underlying the results presented in the study are available from (include the name of the third party*	All XXX files are available from https://github.com/Yingli-C-SYAU/Related-programs-and-code

and contact information or URL).<br>• This text is appropriate if the data are owned by a third party and authors do not have permission to share the data.<br><br>* typeset	
Additional data availability information:	

Powered by Editorial Manager® and ProduXion Manager® from Aries Systems Corporation

Manuscript
Click here to access/download;Manuscript;Inversion Modeling.pdf

Inversion Modeling of Japonica Rice Canopy Chlorophyll Content with UAV Hyperspectral Remote Sensing

Yingli Cao[1,2], Kailun Jiang[1,2], Jingxian Wu[3], Fenghua Yu[1,2], Wen Du[1,2], Tongyu Xu[1,2*]

1 Department of Information and Electrical Engineering, Shenyang Agricultural University, Shenyang 110866, China
2 Liaoning Engineering Research Center for Information Technology in Agriculture, Shenyang 110866, China
3 Department of Electrical Engineering, University of Arkansas, Fayetteville, AR 72701, USA

*yatongmu@163.com

***Abstract*—Chlorophyll content is an important indicator of the growth status of japonica rice. The objective of this paper is to develop an inversion model that can predict japonica rice chlorophyll content by using hyperspectral image of rice canopy collected with unmanned aerial vehicle (UAV). UAV-based hyperspectral remote sensing can provide timely and cost-effective monitoring of chlorophyll content over a large region. The study was based on hyperspectral data collected at the Shenyang Agricultural College Academician Japonica Rice Experimental Base in 2018 and 2019. In order to extract the salient information embedded in the high-dimensional hyperspectral data, we first perform dimension reduction by using a successive projection algorithm (SPA). The SPA extracts the characteristic hyperspectral bands that are used as input to the inversion model. The characteristic bands extracted by SPA are 410 nm, 481 nm, 533 nm, 702 nm, and 798 nm, respectively. The inversion model is developed by using an extreme learning machine (ELM), the parameters of which are optimized by using particle swarm optimization (PSO). The PSO-ELM algorithm can accurately model the nonlinear relationship between hyperspectral data and chlorophyll content. The model achieves a coefficient of determination $R^2 = 0.791$ and a root mean square error of $RMSE = 8.215$. The model exhibits good predictive ability and can provide data support and model reference for research on nutrient diagnosis of japonica rice.**

***Index Terms*—UAV remote sensing; chlorophyll; japonica rice; hyperspectral; extreme learning machine; particle swarm algorithm; successive projection algorithm.**

I. Introduction

Japonica rice is a major Asian rice variety with a large cultivation area in Northeastern China. Japonica rice has a long growth period, high protein content, and good flavor [1]. Chlorophyll content is an important indicator for characterizing the growth status of japonica rice. Non-destructive, rapid, and large-scale estimation of the chlorophyll content of japonica rice has long been an important research direction for precision agriculture [2–5]. Recently there have been growing interests of employing unmanned aerial vehicle (UAV) low-altitude remote sensing platforms in precision agriculture. Compared to other remote sensing methods, UAV remote sensing platforms have the advantages of low cost, high flexibility, large coverage area, and versatility in terms of collected data. This motivates the development of UAV hyperspectral remote sensing platform for rapid assessment of japonica rice chlorophyll content at near-earth scale, which has important practical significance for assisting field precision fertilization and pesticide application.

Hyperspectral data have been used to assess chlorophyll content for a variety of crops [6–9]. In [6], hyperspectral imaging of potato leaves were collected at different vertical leaf positions, and a rational function-partial least squares (RF-PLS) model was developed to estimate the chlorophyll content at different vertical leaf positions by using the hyperspectral data. The results show that the hyperspectral reflectance varies with different vertical leaf positions. In [7], Li et al. studied the reflectance spectrum of winter wheat canopy in the range of 325 to 1075 nm, and a genetic algorithm (GA) was used to optimize the spectral characteristic parameters. In combination with correlation analysis, a least squares-support vector regression method was used to establish a winter wheat chlorophyll content prediction model, which provided support for subsequent fertilization decisions. A UAV low-altitude remote sensing platform was used to collect hyperspectral images of wheat canopy in [8]. A leaf chlorophyll content inversion model was developed by establishing a comprehensive growth index (CGI) during a key wheat production period. The inversion model achieved a coefficient of determination greater than 0.7, which provides a good reference for monitoring wheat chlorophyll content. In [9], hyperspectral data were used to estimate the canopy cholorophyll content of spring wheat by using a partial least squares regression model. It was found that integrating the maximum reflectance of the hyperspectral data from 820 to 940 nm can provide an accurate estimate of the chlorophyll content.

Most current research on crop chlorophyll inversion with hyperspectral data were developed by using statistical regression models, and they can be roughly divided into two categories: vegetation index models [10–12], and direct spectrum models [13, 14]. In the vegetation index models, the hyperspectral data are first used to construct various vegetation indices, which are then used to develop multiple linear or nonlinear regression methods to establish an inversion model between these indices and chlorophyll content. Such an approach has the advantage of simple model constructions with clear physical meanings. However, it requires the construction of a large number of spectral indices in an ad hoc manner, without a systematic guidance to general crop varieties. The various spectral indices depend on the type and region of a given species, thus it lacks universal applicability [10–12]. The direct spectrum models rely on the modeling of the entire hyperspectral bands, which is usually a high dimension vector. Direct utilizing the entire hyperspectral band can results in high model complexity or even model overfitting [13]. This problem can be partially solved by employing dimension reduction techniques, such as principal component analysis (PCA) or PLS [14].

The objective of this work is to develop an inversion model that can predict japonica rice canopy chlorophyll content by using hyperspectral data collected with a UAV remote sensing platform. The study was based on data collected at the Shenyang Agricultural College Academician Japonica Rice Experimental Base in 2018 and 2019. In addition to hyperspectral data of japonica rice canopy collected by UAVs, rice samples were also collected on the ground and analyzed in the lab to provide ground truth for the inversion model. In order to reduce model complexity and avoid model overfitting, we first employ a successive projection algorithm (SPA) to extract the characteristic bands from the high dimension hyperspectral vector. Based on our experiment data, the characteristic bands extracted by SPA are 410 nm, 481 nm, 533 nm, 702 nm, and 798 nm, respectively. With the extracted characteristic bands as input, the inversion model is developed by using an extreme learning machine (ELM). The parameters of ELM, including population size, inertia weight, learning factor, and the velocity position correlation coefficient, are optimized by using particle swarm optimization (PSO). It has been demonstrated that the PSO-ELM outperforms conventional ELM algorithm in terms of the number of hidden layer nodes and network generalizations. The PSO-ELM algorithm accurately models the nonlinear relationship between hyperspectral data and chlorophyll content. The developed model provides a rapid, accurate, and nondestructive approach for chlorophyll diagnosis in japonica rice.

II. Materials and Methods

A. *Experiment Setup*

The experiment was carried out between June and September in 2018 and 2019, respectively. It took place at the Liaozhong Kalima Academician Rice Experimental Station ($41°47'\mathrm{N}, 122°71'\mathrm{E}$) of the Shenyang Agricultural University. The rice strain was “Shendao 529”.

The japonica rice test plots were designed with four different nitrogen fertilizer gradient treatments, namely $\mathrm{CK}, \mathrm{N}_1, \mathrm{N}_2$ and N_3. Each treatment was repeated three times. There were a total of 12 test plots, and the size of each test plot is 30×45 m^2. Among them, CK represents the control group, i.e., no nitrogen fertilizer was applied; N_1 represents the local standard nitrogen fertilizer application level, and the nitrogen fertilizer application amount was 45 kg/ha; N_2 represents the low nitrogen fertilizer application level, and the application amount was half that of N_1; N_3 is the group with the high nitrogen fertilization level, and the application amount was 1.5 times that of N_1. The applications of phosphate fertilizer and potash fertilizer were carried out based on local standard application quantities, that is, 51.75 kg/ha for phosphate fertilizer, and the standard application amount is 18 kg/ha for potassium fertilizer. Fig. 1 shows the design of the test area and test plots for this study.

Fig. 1: Test plots used for data collection.

B. *UAV Hyperspectral Image Data Acquisition*

The UAV hyperspectral platform adopts the M600 PRO six-rotor UAV from Shenzhen Dajiang Innovation Co., Ltd. (Shenzhen, Guangdong, China). The hyperspectral images were collected by using the GaiaSky-mini built-in push-broom airborne hyperspectral imaging system from Sichuan Shuangli Hepu Company (Sichuan, China). The hyperspectral band range is 400 to 1000 nm, with a 3 nm resolution. The total number of effective bands is 253. The hyperspectral imaging system is shown in Fig. 2.

Fig. 2: UAV hyperspectral imaging system

The acquisitions of the hyperspectral images were performed on selected Wednesdays between 11:00 AM and 12:00 PM in the morning. The data were collected on days with sunny or lightly cloudy weather (cloud less than 20%), and the solar light intensity was relatively stable. The flying height of the UAV was a true height of 150 m. The collected hyperspectral data were processed and extracted with the ENVI5.3+IDL software tool. During processing, the spectral angle mapper (SAM) method was first used to remove impacts from interfering objects, then the hyperspectral image was generated by calculating the average spectrum of each area of interest.

C. Ground Data Acquisition and Analysis

In addition to hyperspectral image data, we also performed manual ground data collection in the japonica rice field. Data collected on the ground will serve as ground truth for the UAV hyperspectral data. The ground data cover all six stages of the key growth periods of japonica rice, including tillering, jointing, booting, heading, flowering, and filling. Four japonica rice plants were collected in each test plot, and 50 canopy leaves were placed in a sealed bag and stored in a cooler with temperature around 4°C. The sampled rice plants were located in the middle of the test plots where the growth was even. A hand-held differential GPS device was used to measure the geographical coordinates of the sampling points, which were then correlated with the hyperspectral data collected by the UAV [15–17].

After the japonica rice samples were returned to the laboratory, fully-expanded leaves of the japonica rice samples were selected and cut into small pieces. Pieces from the same leaf are mixed together, and 0.4 g of the leaf mixture was placed in 200 mL of an extraction solution, which contains acetone, ethanol, and distilled water at a ratio of 9:9:2. The solutions with leaf mixture are left to stand in the shady area of a laboratry with temperature around 20°C. Once the leaf sample appeared to be completely white, colorimetry was then performed with a spectrophotometer to measure the optical density (OD) at 663 nm and 645 nm, respectively. Based on the colorimetry result, the chlorophyll content of the japonica rice samples was calculated according to equation (1) [18]

$$\texttt{Chl}(\text{mg/L}) = 5.134\texttt{OD}_{663} + 20.436\texttt{OD}_{645} \quad (1)$$

where $\texttt{Chl}$ is the chlorophyll level, and $\texttt{OD}_{663}$ and $\texttt{OD}_{645}$ are the ODs at 663 nm and 645 nm, respectively.

A total of 10 experimental data collections were conducted in this study, and 196 valid data samples were collected. Of these, 84% were selected to form a modeling dataset (165 samples), and the remaining 16% were used to construct a validation dataset (31 samples). The statistical characteristics of the sampled data are shown in TABLE I.

TABLE I: Statistical characteristics of chlorophyll content in japonica rice in test plots

Sample set	Number of samples	Chlorophyll content maximum (mg/L)	Chlorophyll content minimum (mg/L)	Chlorophyll content average (mg/L)	Standard deviation (mg/L)	Coefficient of variation (%)
Overall	196	99.70	2.60	54.66	26.94	49.28
Modeling dataset	165	99.70	2.60	54.33	27.31	50.27
Validation dataset	31	93.80	10.70	56.42	25.22	44.70

According to TABLE I, the modeling and validation dataset share similar statistical properties with the exception of sample size. Additionally, the coefficient of variation was greater than 40%, indicating that the chlorophyll content data exhibited high dispersion.

Fig. 3: Global sensitivity analysis of the input parameters in the PROSPECT model

D. Extraction of characteristic hyperspectral bands

The successive projection algorithm (SPA) was used to extract hyperspectral information from characteristic spectral bands of the japonica rice canopy. Information obtained from SPA was then used as input to the chlorophyll content inversion model. Previous research by our group verified that the change in chlorophyll content of japonica rice mainly affects the spectral reflectance between 400 to 800 nm (Fig. 3). The detailed analysis method is described in [19, 20]. Therefore, 400 to 800 nm was selected as the hyperspectral interval in this study.

SPA is a forward variable selection algorithm that minimizes collinearity in a vector space. Define a size $M \times J$ calibration data matrix as $\mathbf{X}_{\text{cal}} = [\mathbf{x}_1, \cdots, \mathbf{x}_J]$, where J is the number of candidate wavelengths, M is the number of calibration waveforms from the hyperspectral image, and $\mathbf{x}_j$ is a length M vector. The objective of SPA is to identify a subset of $N < J$ columns of $\mathbf{X}_{\text{cal}}$ with minimum collinearity, where N is a predefined parameter. Such an approach can identify a subset of wavelengths with minimum redundant information.

SPA performs forward subset selection in an iterative manner. It starts with one wavelength, and then add a new wavelength at each iteration until N wavelengths are selected. Define the set of selected wavelengths as $\mathcal{A}$, and the set of unselected wavelengths as $\mathcal{S}$, such that $\mathcal{A} \bigcup \mathcal{S} = \mathcal{J} = \{1, \cdots, J\}$.

During the iterative selection process, we will project each vector with wavelength in $\mathcal{S}$ onto the subspace spanned by the vectors indexed by the elements in $\mathcal{A}$. The vector with the largest residue after projection contains the most new information that is not represented by the elements in $\mathcal{A}$, thus the corresponding wavelength will be added to $\mathcal{A}$.

The initial values of $\mathcal{A}$ and $\mathcal{S}$ are $\mathcal{A} = \emptyset$ and $\mathcal{S} = \mathcal{J}$, respectively, where $\emptyset$ is the empty set. At the beginning of the iteration, assign $\mathbf{y}_j = \mathbf{x}_j$, for all $j \in \mathcal{S}$. The values of $\mathbf{y}_j$ will be iteratively updated throughout the iteration process. Denote the wavelength selected at the k-th iteration as a_k. The corresponding vector with index a_k is represented as $\mathbf{z}_k = \mathbf{y}_{a_k}$. Each iteration contains the following three steps [21, 22].

Step 1 (Projection). During the k-th iteration, we first project all vectors $\mathbf{y}_j$, $j \in \mathcal{S}$, into the null space of $\mathbf{z}_{k-1}$ as

$$\mathbf{e}_j = \begin{cases} \mathbf{y}_j - \mathbf{z}_{k-1}(\mathbf{z}_{k-1}^T \mathbf{z}_{k-1})^{-1} \mathbf{z}_{k-1}^T \mathbf{y}_j, & k > 1, \\ \mathbf{y}_j, & k = 1, \end{cases} \quad \forall j \in \mathcal{S} \tag{2}$$

For the first iteration with $k = 1$, since $\mathcal{A}$ is empty, the projection operator to the null space of the vectors indexed by $\mathcal{A}$ is simply the size M identity matrix $\mathbf{I}_M$.

Step 2 (Selection). The index of the residual vector $\mathbf{e}_j$ with the largest ℓ_2 norm is selected as the new wavelength as

$$a_k = \underset{j \in \mathcal{S}}{\operatorname{argmax}} \|\mathbf{e}_j\|_2 \tag{3}$$

Step 3 (Update). Update the sets $\mathcal{A} = \mathcal{A} \bigcup \{a_k\}$ and $\mathcal{S} = \mathcal{S} \backslash \{a_k\}$, where the operator $\backslash$ represents the difference between two sets. Update all $\mathbf{y}_j$ as the residual of the projection as

$$\mathbf{y}_j = \mathbf{e}_j, \text{ for all } j \in \mathcal{S}. \tag{4}$$

The output of SPA is the set of wavelength indices $\mathcal{A}$. To further reduce the number of wavelengths, multiple linear regression (MLR) models were applied to all subsets of $\mathcal{A}$. The subset that results in the smallest root mean square error (RMSE) is selected as effective characteristic hyperspectral bands.

E. PSO-ELM inversion modeling of chlorophyll content

The inversion model of japonica rice canopy chlorophyll content was established by using the subset of characteristic hyperspectral bands obtained from the previous section. The relationship between the chlorophyll content and the characteristic hyperspectral bands were modeled by using extreme learning machine (ELM) with particle swarm optimization (PSO). The PSO algorithm was used to optimize the selection of input layer weights and hidden layer biases of the ELM to calculate the output weight matrix. The parameters of the PSO algorithm considered in this study mainly include population size ($\mathtt{pop}$), inertia weight ($\mathtt{w}$), learning factor ($\mathtt{C}_1, \mathtt{C}_2$), and the velocity position correlation coefficient ($\mathtt{m}$). It has been demonstrated that the PSO-ELM outperforms conventional ELM algorithm in terms of the number of hidden layer nodes and network generalizations [23]. In this study, the accuracy of the inversion model is assessed by using the RMSE and the coefficient of determination (R^2).

III. Results and analysis

A. Extraction of characteristic hyperspectral band

Fig. 4: Noise reduction of hyperspectral information smoothing

We first study the correlation between the japonica rice chlorophyll content and the various hyperspectral bands from the japonica rice canopy reflectance data. Three methods, including Pearson's correlation coefficient, distance correlation coefficient, and maximum information coefficient (MIC), were used to analyze the correlation between chlorophyll and each individual band. Before applying the correlation methods, the Savitzky-Golay convolution smoothing algorithm was used to smooth the 400 to 800 nm reflectance spectral data, obtained from the test plots and reduce the effect of noise on chlorophyll content inversion accuracy. The japonica rice canopy hyperspectral curve after noise reduction is shown in Fig. 4.

Results from the correlation analysis are shown in Fig. 5. Results from all three methods in general have similar trends: the maximum correlation between chlorophyll content and hyperspectral reflectance is achieved in the wavelength range between 701 to 705 nm. Specifically, the peak correlation is located at the wavelength of 702 nm for all three methods, with the distance, Pearson and MIC correlation coefficients being 0.66, 0.64, and 0.53, respectively. In addition to the maximum correlation band, relatively strong correlations are also observed at other bands, such as the band with wavelength between 410 to 480 nm.

The SPA algorithm described in Section II-D was used to extract characteristic hyperspectral bands for the chlorophyll content inversion model. Based on our experiment data, the extracted characteristic wavelengths were 410 nm, 481 nm, 533 nm, 702 nm, and 798 nm. Fig. 6 shows the average reflectance marked with the extracted characteristic bands.

Fig. 5: Single band correlation analysis results

Fig. 6: Selection of characteristic bands of SPA in the canopy layer of japonica rice

B. PSO-ELM chlorophyll content results

The inversion model of cholorophyll content is studied in this subsection. In the inversion model, the reflectance data on the characteristic hyperspectral bands extracted from SPA are used as independent variables. The chlorophyll contents obtained through ground data acquisition constitute the dependent variables. The inversion model was established by using PSO-ELM. For comparison, we also include the results obtained from conventional ELM. In PSO-ELM, the number of particle swarm iterations was 100. The chlorophyll contents predicted by hyperspectral data with the inversion model are compared to those obtained from ground data, and the performance is evaluated by using the coefficient of determination (R^2).

Due to the large number of parameters of the PSO algorithm, an orthogonal array test was designed to identify the optimum PSO parameter settings. The results are shown in TABLE II, where each parameter can take one of 5 values, and there are 5 parameters.

TABLE II: Orthogonal array test for PSO-ELM

No	Model	pop	w	C_1	C_2	m	R^2
1	PSO-ELM	40	0.3	1.1	1.1	0.02	0.721
2	PSO-ELM	40	0.9	1.3	1.3	0.06	0.763
3	PSO-ELM	40	1.5	1.65	1.65	0.2	0.753
4	PSO-ELM	40	0.9∼0.3	2.8	2.8	0.6	0.773
5	PSO-ELM	40	0.3∼1.5	3.5	3.5	1	0.772
6	PSO-ELM	50	0.3	1.3	1.65	0.6	0.773
7	PSO-ELM	50	0.9	1.65	2.8	1	0.758
8	PSO-ELM	50	1.5	2.8	3.5	0.02	0.759
9	PSO-ELM	50	0.9∼0.3	3.5	1.1	0.06	0.757
10	PSO-ELM	50	0.3∼1.5	1.1	1.3	0.2	0.773
11	PSO-ELM	60	0.3	1.65	3.5	0.06	0.761
12	PSO-ELM	60	0.9	2.8	1.1	0.2	0.765
13	PSO-ELM	60	1.5	3.5	1.3	0.6	0.721
14	PSO-ELM	60	0.9∼0.3	1.1	1.65	1	0.773
15	PSO-ELM	60	0.3∼1.5	1.3	2.8	0.02	0.746
16	PSO-ELM	70	0.3	2.8	1.3	1	0.739
17	PSO-ELM	70	0.9	3.5	1.65	0.02	0.779
18	PSO-ELM	70	1.5	1.1	2.8	0.06	0.687
19	PSO-ELM	70	0.9∼0.3	1.3	3.5	0.2	0.773
20	PSO-ELM	70	0.3∼1.5	1.65	1.1	0.6	0.773
21	PSO-ELM	80	0.3	3.5	2.8	0.2	0.773
22	PSO-ELM	80	0.9	1.1	3.5	0.6	0.762
23	PSO-ELM	80	1.5	1.3	1.1	1	0.733
24	PSO-ELM	80	0.9∼0.3	1.65	1.3	0.02	0.760
25	PSO-ELM	80	0.3∼1.5	2.8	1.65	0.06	0.760
	ELM						0.667

TABLE III summarizes the orthogonal array test results of the PSO-ELM parameters, where W_{ij} is the sum of R^2 obtained from the i-th value of parameter j. For example, W_{11} is obtained by calculating the sum of R^2 corresponding to the first value of the first parameter, pop $= 40$, and W_{12} is obtained by calculating the sum of R^2 corresponding to the first value of the second parameter, $\mathtt{w} = 0.3$, as

$$\mathrm{W}_{11} = \mathrm{R}_1^2 + \mathrm{R}_2^2 + \mathrm{R}_3^2 + \mathrm{R}_4^2 + \mathrm{R}_5^2 \tag{5}$$

$$\mathrm{W}_{12} = \mathrm{R}_1^2 + \mathrm{R}_6^2 + \mathrm{R}_{11}^2 + \mathrm{R}_{16}^2 + \mathrm{R}_{21}^2 \tag{6}$$

TABLE III: Analysis of the PSO-ELM orthogonal array test results

	pop	w	C_1	C_2	m
W_{1j}	3.782	3.767	3.716	3.749	3.765
W_{2j}	**3.820**	3.827	3.788	3.756	3.728
W_{3j}	3.766	3.653	**3.805**	**3.838**	**3.837**
W_{4j}	3.751	3.824	3.796	3.737	3.802
W_{5j}	3.788	**3.836**	3.802	3.827	3.775

Other values of W_{ij} are calculated in a similar manner, and the results are shown in TABLE III. Each column of TABLE III indicates the impacts of one parameter on the perdiction accuracy. For example, on the first column, the differences among W_{i1}, for $i = 1, \cdots, 5$, reveal how the choice of population size affect the accuracy of the inversion model, regardless the values of other parameters. Since $W_{21} \geq W_{i1}$, it is concluded that the population size that maximize R^2 shoud be the 2th value in the orthogonal array test, that is, $\texttt{pop} = 50$. Similary, the columns corresponding to $\texttt{w}$, $\texttt{C}_1$, $\texttt{C}_2$, and $\texttt{m}$ are maximized at W_{52}, W_{33}, W_{34}, and W_{35}, respectively. Consequently, the optimum values of the parameters are $\texttt{pop} = 50$, $\texttt{w} = 0.9 \sim 0.3$, $\texttt{C}_1 = 1.65$, $\texttt{C}_2 = 2.8$, and $\texttt{m} = 0.2$.

With the set of parameters obtained through the orthogonal array test, we can obtain the PSO-ELM japonica rice chlorophyll content inversion model. Applying the newly developed inversion model to the experiment data, we obtain $R^2 = 0.791$ and RMSE $= 8.215$. These results are much better than the conventional ELM model, which obtains $R^2 = 0.667$ and RMSE $= 11.308$. The inversion effect is shown in Fig. 7. From the inversion results, it can be seen that the PSO-ELM used in this study demonstrates significantly better prediction performance for chlorophyll content than the model established by the conventional ELM method.

Fig. 7: Inversion results of Japonica rice canopy chlorophyll content

IV. Discussion

We have built an inversion model that can estimate the japonica rice chlorophyll content by using hyperspectral data of japonica rice canopy collected by UAVs. The inversion model enables rapid monitoring and assessment of japonica rice chlorophyll content with UAV remote sensing. Compared to existing methods, the proposed inversion model has two main contributions.

First, an efficient dimension reduction method with SPA was employed to extract the salient information embedded in the high dimensional hyperspectral data. The SPA-based dimension reduction approach provides a more efficient and accurate representation of the hyperspectral contents than the conventional one-dimensional or multiple-regression statistical models, which may not fully express the nonlinear relationship between spectral information and chlorophyll contents [24–27]. The subset of the characteristic hyperspectral band extracted by the SPA algorithm are used as the input of the inversion model.

Second, the inversion model was constructed by using the PSO-ELM algorithm. Conventional ELM algorithm suffers from poor generalization ability and low calibration accuracy due to random input weights and hidden layer thresholds when there are fewer hidden layer nodes. To solve these problems, the PSO algorithm provides a systematic way to optimize input weights and hidden layer thresholds, thus renders a much better model accuracy compared to conventional ELM algorithms.

Comment 1: characteristic bands. The characteristic bands selected in this study are in general the same as those used in previous studies in the characteristic band range, but the specific characteristic bands differ [28–33]. The difference in the specific characteristic bands is mainly due to the following factors: 1) the rice variety used in this study is different from previous works; and 2) the hyperspectral information might be affected by the presence of water, weeds, and soil in rice fields.

Comment 2: future improvements. The accuracy of the inversion model can be improved by increasing the amount of data collected from both the UAV platform and ground data. More data can reduce the uncertainties caused by noise, interference, and inevitable collection errors. In addition, the inversion model was established only for experimental japonica rice varieties. Future research will focus on increasing the number of test species and establishing chlorophyll content inversion models for different growth stages of japonica rice in order to improve model accuracy and universal applicability.

V. Conclusions

We have developed an inversion model that can predict japonica rice chlorophyll content by using rice canopy hyperspectral data collected from UAV platforms. The model was developed by using data collected from the Shendao 529 rice variety with both UAV and ground measurement performed in 2016 and 2017. The inversion model was developed by using a combination of the SPA method and the PSO-ELM algorithm. Specifically, the SPA method was used to achieve hyperspectral data dimension reduction by extracting the hyperspectral characteristic bands, which were then used used as the input to the PSO-optimized ELM algorithm to establish the nonlinear relationship between the chlorophyll content and hyperspectral data. Based on the experimental results, we have the following conclusions.

1) The SPA algorithm is effective in extracting hyperspectral characteristic bands in the range of 400 to 800 nm. The characteristic hypersepctral bands selected by the SPA algorithm were 410 nm, 481 nm, 533 nm, 702 nm, and 798 nm, respectively.

2) The proposed inversion model achieves $R^2 = 0.791$ and RMSE $= 8.215$, which are significantly better than the inversion model of the conventional ELM algorithm with $R^2 = 0.667$ and RMSE $= 11.308$. The model exhibits good predictive ability and can provide data support and model reference for Northeast japonica rice chlorophyll content research and nutrient diagnosis.

Funding

The work for YC and JK was supported by National Key R&D Program of China (2016YFD0200700).

Authors' contributions

YC and JK designed the experiment and collected all the data. FY, WD, JW, and YC designed and implemented the algorithm based on the idea of TX. All authors read and approved the final manuscript.

Acknowledgements

We greatly thank Dr. Dianrong Ma and Minghui Zhao at Shenyang Agricultural College Academician Japonica Rice Experimental Base for providing experimental fields and cultivating the paddies.

References

[1] Gao Jiping, Sui Yanghui, ZHANG Wenzhong,et al. Effects of Biochar on Seedling Growth and Endogenous Hormone of Rice [J]. Journal of Shenyang Agricultural University,2017,48(2):145-151.
[2] Romero M, Luo Y, Su B, et al. Vineyard water status estimation using multispectral imagery from an UAV platform and machine learning algorithms for irrigation scheduling management [J]. Computers & Electronics in Agriculture, 2018, 147:109-117.
[3] Kong W, Huang W, Casa R, et al. Off-Nadir Hyperspectral Sensing for Estimation of Vertical Profile of Leaf Chlorophyll Content within Wheat Canopies: [J]. Sensors, 2017, 17(12):2711.
[4] Feng Shuai,Xu Tongyu,Yu Fenghua,et al. Research of Method for Inverting Nitrogen Content in Canopy Leaves of Japonica Rice in Northeastern China Based on Hyperspectral Remote Sensing of Unmanned Aerial Vehicle [J]. Spectroscopy and Spectral Analysis, 2019,39(10):3281-3287. (in Chinese with English Abstract)
[5] Zhu Mengyuan,Yang Hongbing,Li Zhiwei. Early Detection and Identification of Rice Sheath Blight Disease Based on Hyperspectral Image and Chlorophyll Content [J]. Spectroscopy and Spectral Analysis, 2019,39(06):1898-1904. (in Chinese with English Abstract)
[6] Sun Hong, Zheng Tao, Liu Ning, Cheng Meng, Li Minzan, Zhang Qin. Vertical distribution of chlorophyll in potato plants based on hyperspectral imaging [J]. Transactions of the Chinese Society of Agricultural Engineering (Transactions of the CSAE), 2018, 34(1): 149-156. (in Chinese with English abstract).
[7] Mao Bohui, Li Minzan, Sun Hong, Liu Haojie, Zhang Junyi, Zhang Qin. Optimization of spectroscopy parameters and prediction of chlorophyll content at seeding stage of winter wheat [J]. Transactions of the Chinese Society of Agricultural Engineering (Transactions of the CSAE), 2017, 33(Supp.1): 164-169. (in Chinese with English abstract).
[8] Pei Haojie, Feng Haikuan, Li Changchun, Jin Xiuliang, Li Zhenhai, Yang Guijun. Remote sensing monitoring of winter wheat growth with UAV based on comprehensive index [J]. Transactions of the Chinese Society of Agricultural Engineering (Transactions of the CSAE), 2017, 33(20): 74-82. (in Chinese with English abstract)
[9] Nijat Kasim, Shi Qingdong, Wang Jingzhe, Rukeya Sawut, Ilyas Nurmemet, Gulnur Isak. Estimation of spring wheat chlorophyll content based on hyperspectral features and PLSR model [J]. Transactions of the Chinese Society of Agricultural Engineering (Transactions of the CSAE), 2017, 33(22): 208-216. (in Chinese with English abstract).
[10] Yu Fenghua, Xu Tongyu, Du Wen, Ma Hang, Zhang Guosheng, Chen Chunling. Radiative transfer models (RTMs) for field phenotyping inversion of rice based on UAV hyperspectral remote sensing, International Journal of Agricultural and Biological Engineering, 2017.7, 10(4): 150 157.
[11] Yu F H, Xu T Y, Cao Y L, Yang G J, Du W, Wang S. Models for estimating the leaf NDVI of japonica rice on a canopy scale by combining canopy NDVI and multisource environmental data in Northeast China [J]. Int J Agric & Biol Eng, 2016; 9(5): 132-142.
[12] Jiang Hailing, Yang Hang, Chen Xiaoping, et al. Research on accuracy and stability if inversing vegetation chlorophyll content by spectral index method [J]. Spectroscopy and Spectral Analysis, 2015, 35(4): 975-981. (in Chinese with English abstract)
[13] Kjær A, Nielsen G, Stærke S, et al. Detection of Glycoalkaloids and Chlorophyll in Potatoes by Hyperspectral Imaging [J]. American Journal of Potato Research, 2017, 94(6):1-10.
[14] Yao Yunjun, Qin qiming, Zhang Zili, et al. Research progress of hyperspectral technology applied in agricultural remote sensing [J]. Transactions of the Chinese Society of Agricultural Engineering (Transactions of the CSAE), 2008, 24(7): 301-306. (in Chinese with English abstract)
[15] Niu Qinglin, Feng Haikuan, Yang Guijun, et,al. Monitoring plant height and leaf area index of maize breeding material based on UAV digital images [J]. Transactions of the Chinese Society of Agricultural Engineering (Transactions of the CSAE), 2018, 34(5): 73-82. (in Chinese with English abstract).
[16] Zhao Xiaoqing, Yang Guijun, et,al. Estimation of soybean breeding yield based on optimization of spatial scale of UAV hyperspectral image [J]. Transactions of the Chinese Society of Agricultural Engineering (Transactions of the CSAE), 2017, 33(1): 110-116. (in Chinese with English abstract).
[17] Pei Haojie, Feng Haikuan, Li Changchun, et, al. Remote sensing monitoring of winter wheat growth with UAV based on comprehensive index [J]. Transactions of the Chinese Society of Agricultural Engineering (Transactions of the CSAE), 2017, 33(20): 74-82. (in Chinese with English abstract)
[18] Xie Kai, Jiang Ping, Luo Yahui. Correlation between chlorophyll content and spectral characteristics of rice leaves under Rice Blast [J]. Chinese Agricultural Science Bulletin, 2017, 33(17):117-122.
[19] Yu Fenghua, Xu Tongyu, Du Wen, Ma Hang, Zhang Guosheng, Chen Chunling. Radiative transfer models (RTMs) for field phenotyping inversion of rice based on UAV hyperspectral remote sensing, International Journal of Agricultural and Biological Engineering, 2017.7, 10(4): 150 157.
[20] Wang Yang, Xiao Wen, Zhou Huancheng, et al. Plant Leaf Dry Matter Estimating and Modeling Based on PROSPECT Model [J]. Journal of Shenyang Agricultural University, 2018, 49(01): 121- 127.
[21] De V A, De A A G, De D S F, et al. Vis-NIR spectrometric determination of Brix and sucrose in sugar production samples using kernel partial least squares with interval selection based on the successive projections algorithm [J]. Talanta, 2018, 181:38-43.
[22] Liu Mingbo, Tang Yanlin, Li Xiaoli,et,al. Feasibility of using successive projections algorithm in spectral monitoring of rice leaves nitrogen contents [J]. Infrared and Laser Engineering, 2014,43(04):1265-1271.
[23] Xiao Huaichun, Sun Xudong, Zhu Danning, et,al. Spectral feature selection and discriminant model building for citrus leaf Huanglongbing [J]. Transactions of the Chinese Society of Agricultural Engineering (Transactions of the CSAE), 2018, 34(3): 180 -187. (in Chinese with English abstract)
[24] Xiao Huaichun, Sun Xudong, Zhu Danning, et,al. Spectral feature selection and discriminant model building for citrus leaf Huanglongbing [J]. Transactions of the Chinese Society of Agricultural Engineering (Transactions of the CSAE), 2018, 34(3): 180 -187. (in Chinese with English abstract)
[25] Sun Y, Wang Y, Xiao H, et al. Hyperspectral imaging detection of decayed honey peaches based on their chlorophyll content [J]. Food Chemistry, 2017, 235:194-202.
[26] Irteza S M, Nichol J E. Measurement of Sun Induced Chlorophyll Fluorescence Using Hyperspectral Satellite Imagery [J]. ISPRS - International Archives of the Photogrammetry, Remote Sensing and Spatial Information Sciences, 2016, 8:911-913.
[27] Fu Yuanyuan, Wang Jihua, Yang Guijun, et al. Band depth analysis and partial least square regression based winter wheat biomass estimation using hyperspectral measurements [J]. Spectroscopy and Spectral Analysis, 2013, 32(5): 1315-1319. (in Chinese with English Abstract)
[28] SUN Gang, HUANG Wenjiang, CHEN Pengfei, et,al. Advances in UAV-based Multispectral Remote Sensing Applications [J].Transactions of the Chinese Society for Agricultural Machinery,2018,49(3):1-17.
[29] Zhang Jian, Meng Jin, Zhao Biquan, et al. Research on the Chlorophyll Content (SPAD) Distribution Based on the Consumer-Grade Modified Near-Infrared Camera [J]. Spectroscopy and Spectral Analysis, 2018,38(03):737-744. (in Chinese with English Abstract)
[30] Liu Ke, Lu Jian, Gao Mengtao, et,al. Effects of Different Nitrogen Treatments on the Relationship Between Flag Leaves Spectral Characteristic and SPAD and IPAR in Hybrid Rice Varieties [J]. Journal of nuclear agricultural sciences. 2018, 32(02):362-369.
[31] Zhang Jian, Li Yong, Xie Jing, et al. Research on Optimal Near-Infrared Band Selection of Chlorophyll (SPAD) 3D Distribution about Rice [J]. Spectroscopy and Spectral Analysis, 2017,37(12):3749-3757. (in Chinese with English Abstract)
[32] Xu Jin,Meng Jihua. Overview on Estimating Crop Chlorophyll Content with Remote Sensing [J]. Remote Sensing Technology and Application, 2016, 31(1): 74-85.
[33] Zhang Jinheng, Wang Juan, Han Chao. Prediction of Chlorophyll Concentration of Rice Based on Spectral Absorption, Transmittance and Reflectance [J]. Chinese Agricultural Science Bulletin. 2010, 26(07):78-83.

Fig. 1: Test plots used for data collection

Click here to access/download;Figure;Fig1.eps

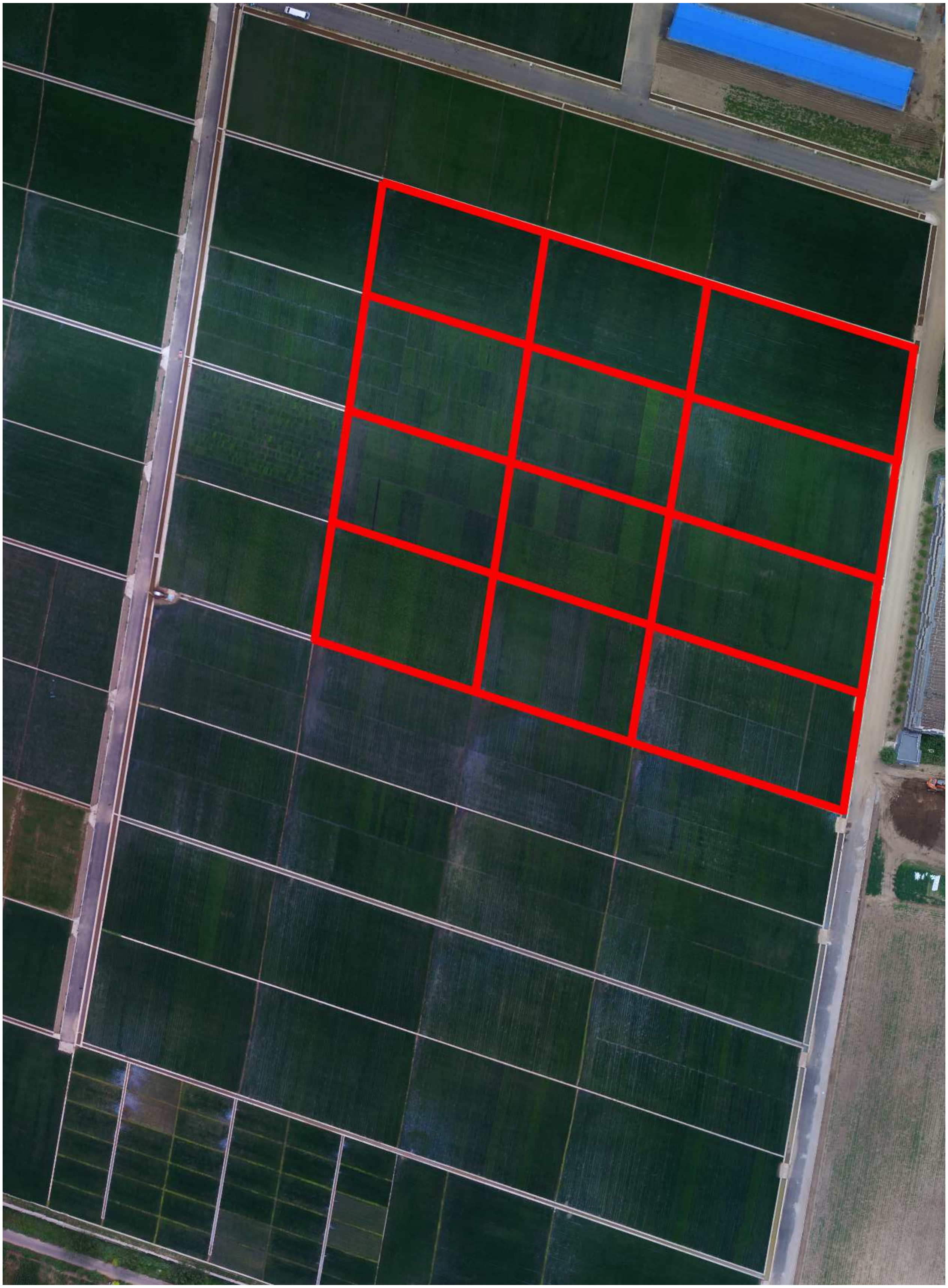

Fig. 2: UAV hyperspectral imaging system

Click here to access/download;Figure;Fig2.eps

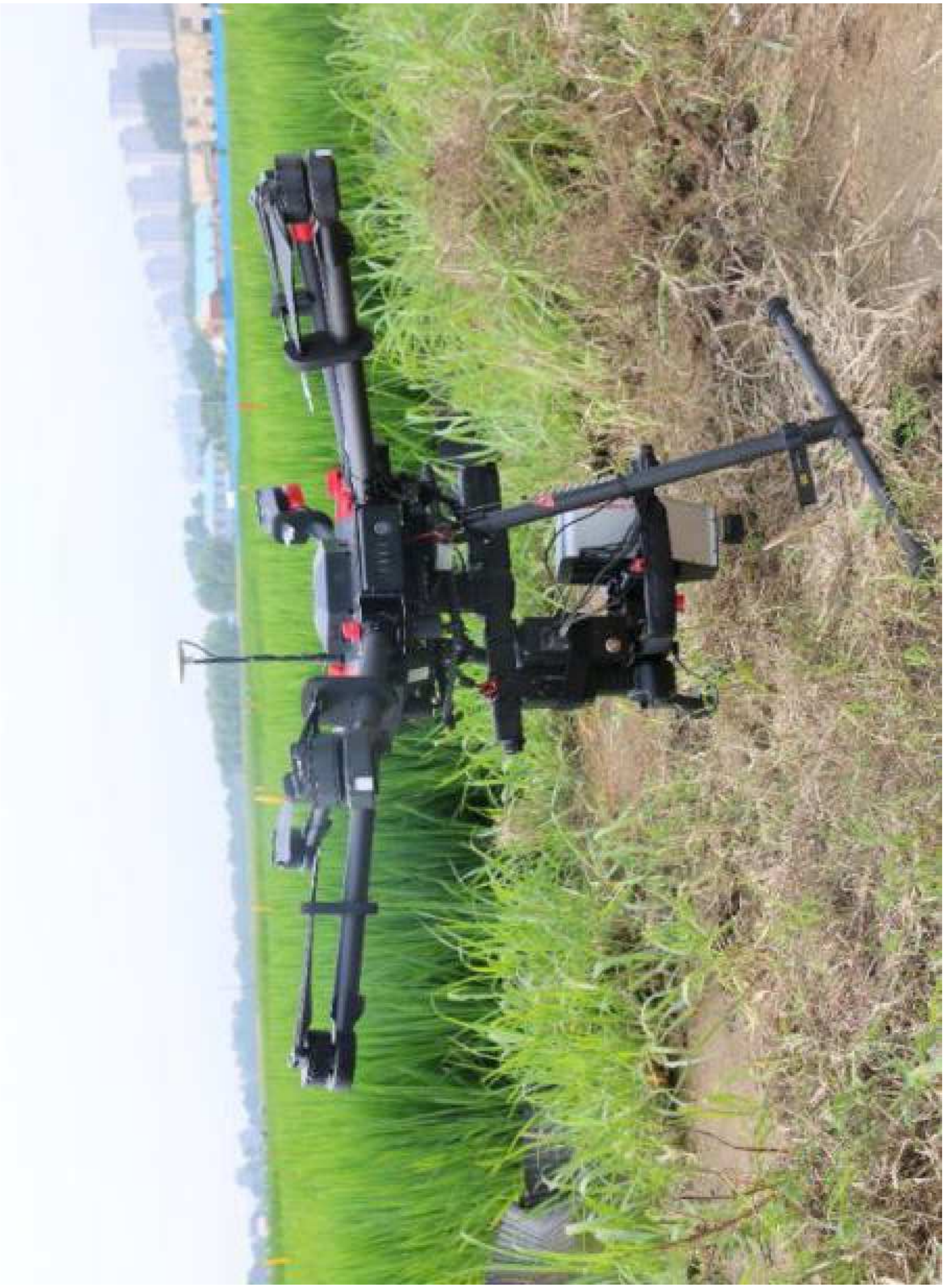

Fig. 3: Global sensitivity analysis

Click here to access/download;Figure;Fig3.eps

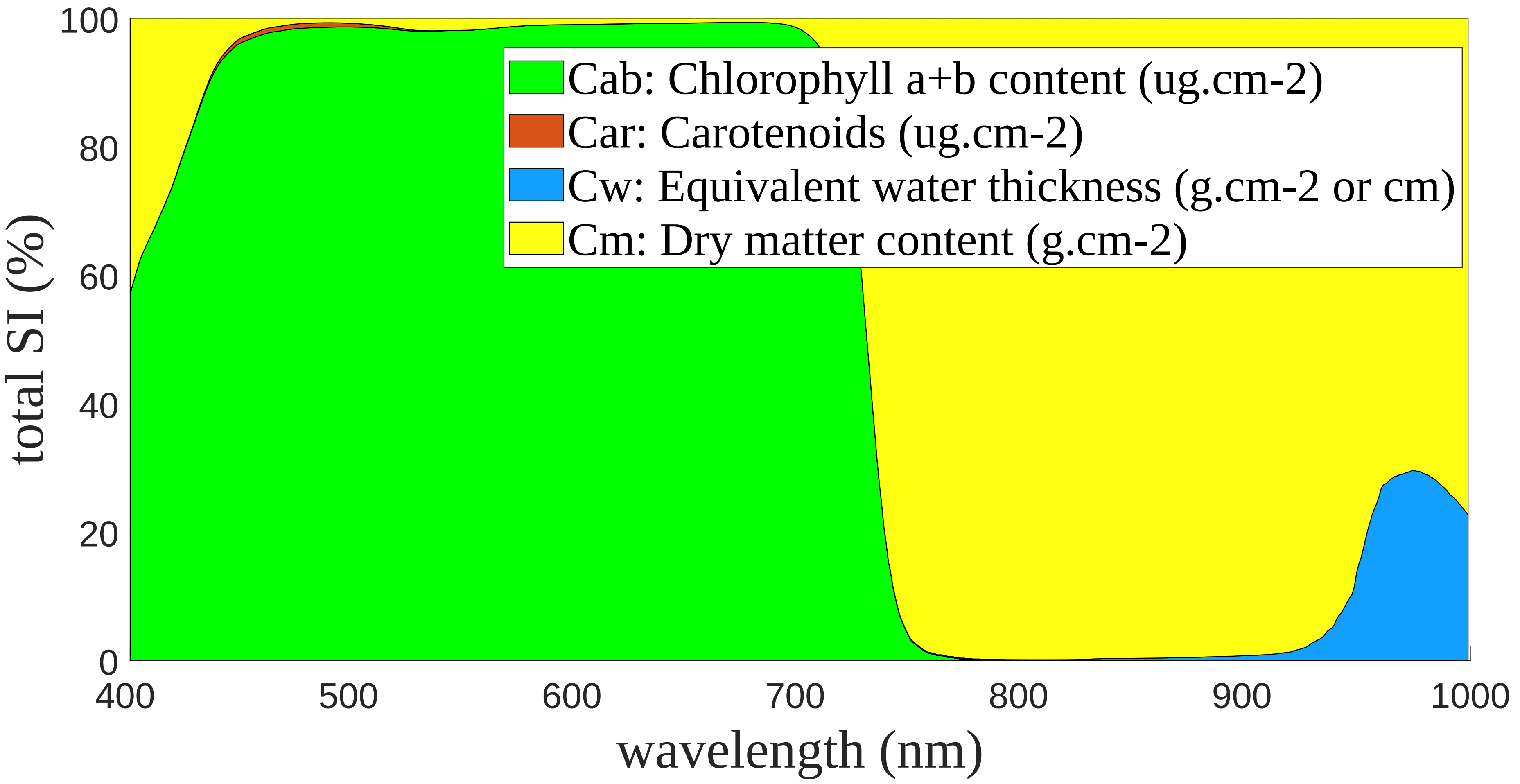

Fig. 4: Noise reduction of hyperspectral

Click here to access/download;Figure;Fig4.eps

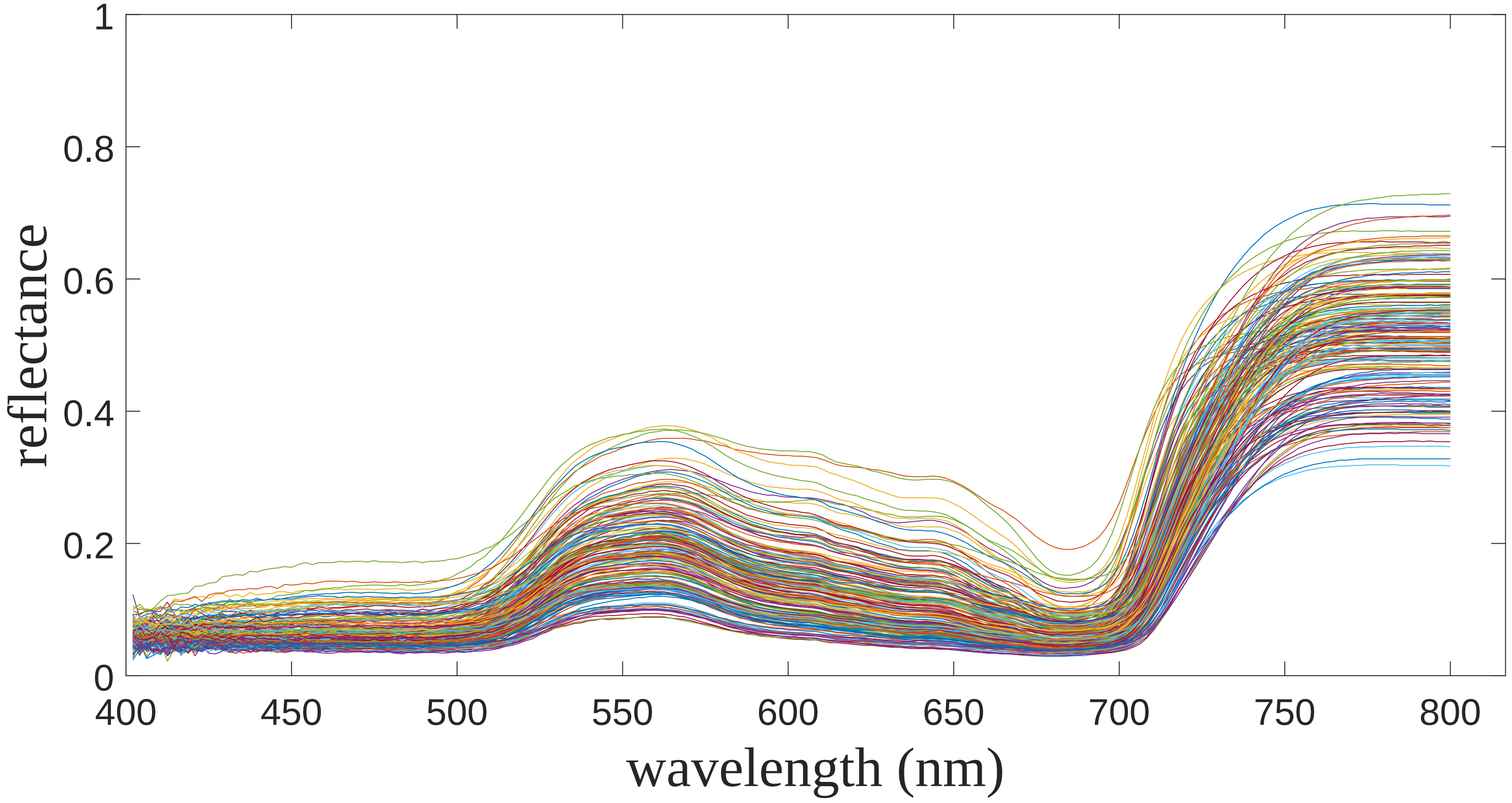

Fig. 5: Single band correlation analysis

Click here to access/download;Figure;Fig5.eps

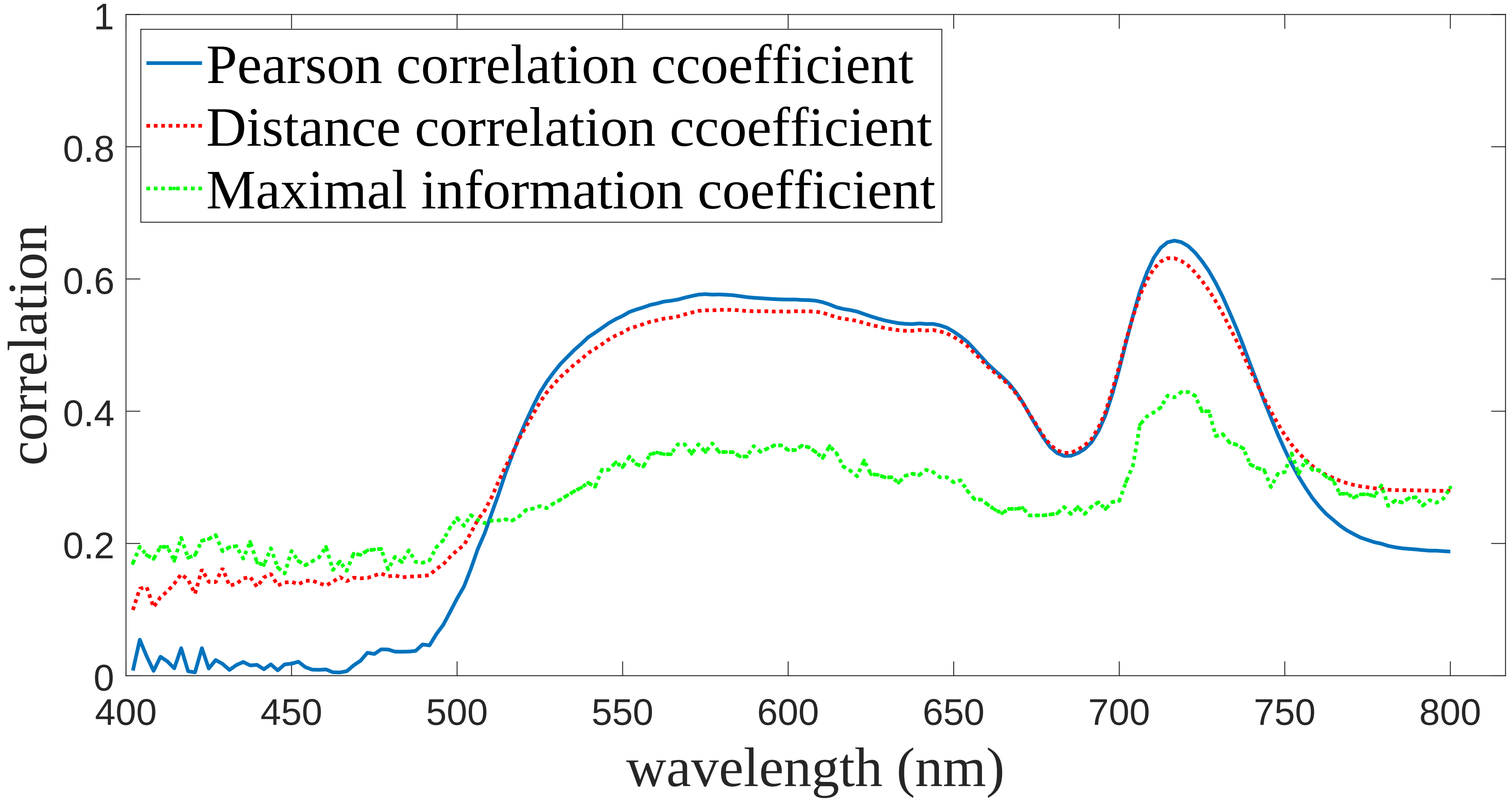

Fig. 6: Selection of characteristic bands of SPA

Click here to access/download;Figure;Fig6.eps

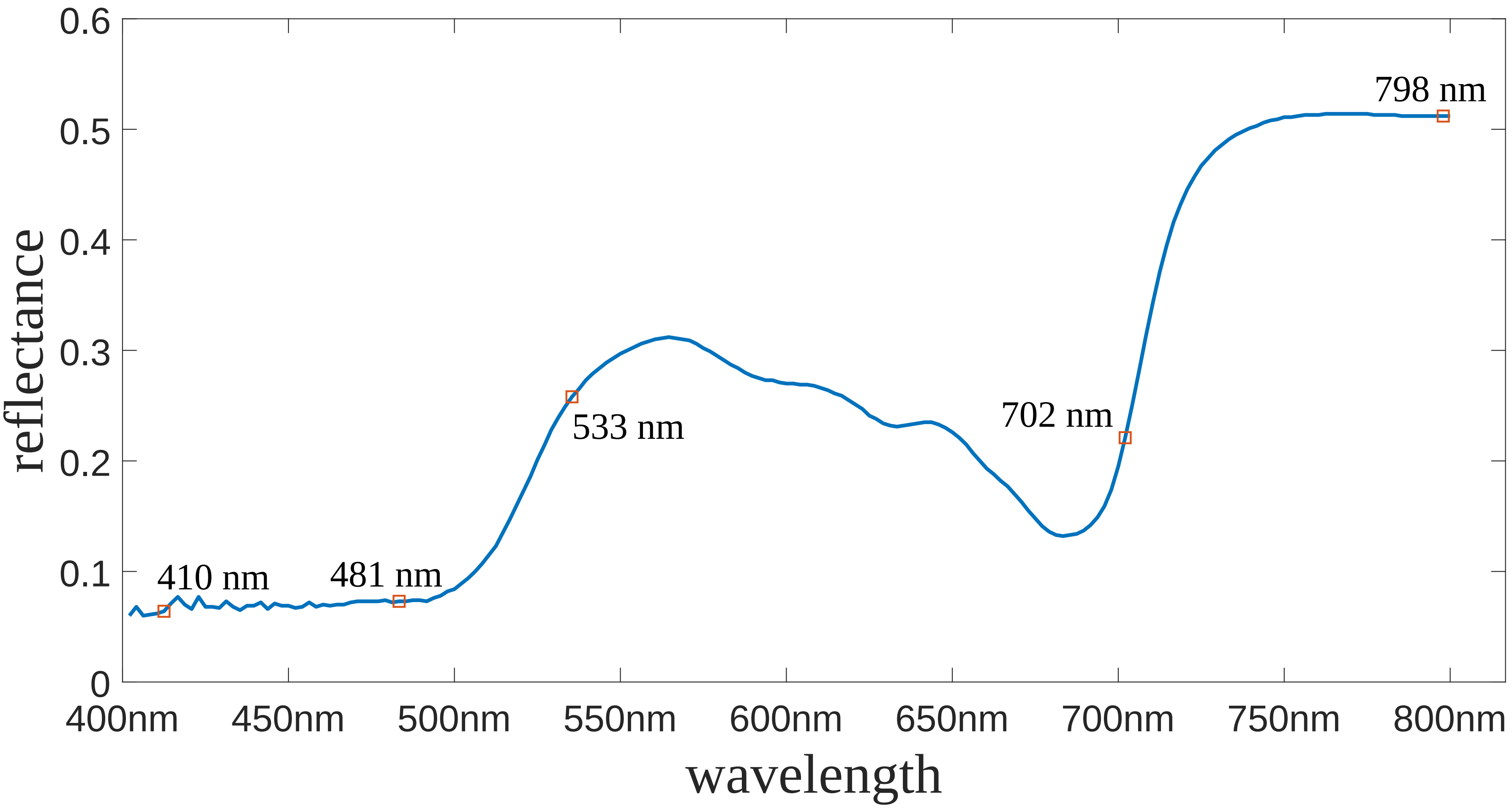

Fig. 7: Inversion results

Click here to access/download;Figure;Fig7.eps

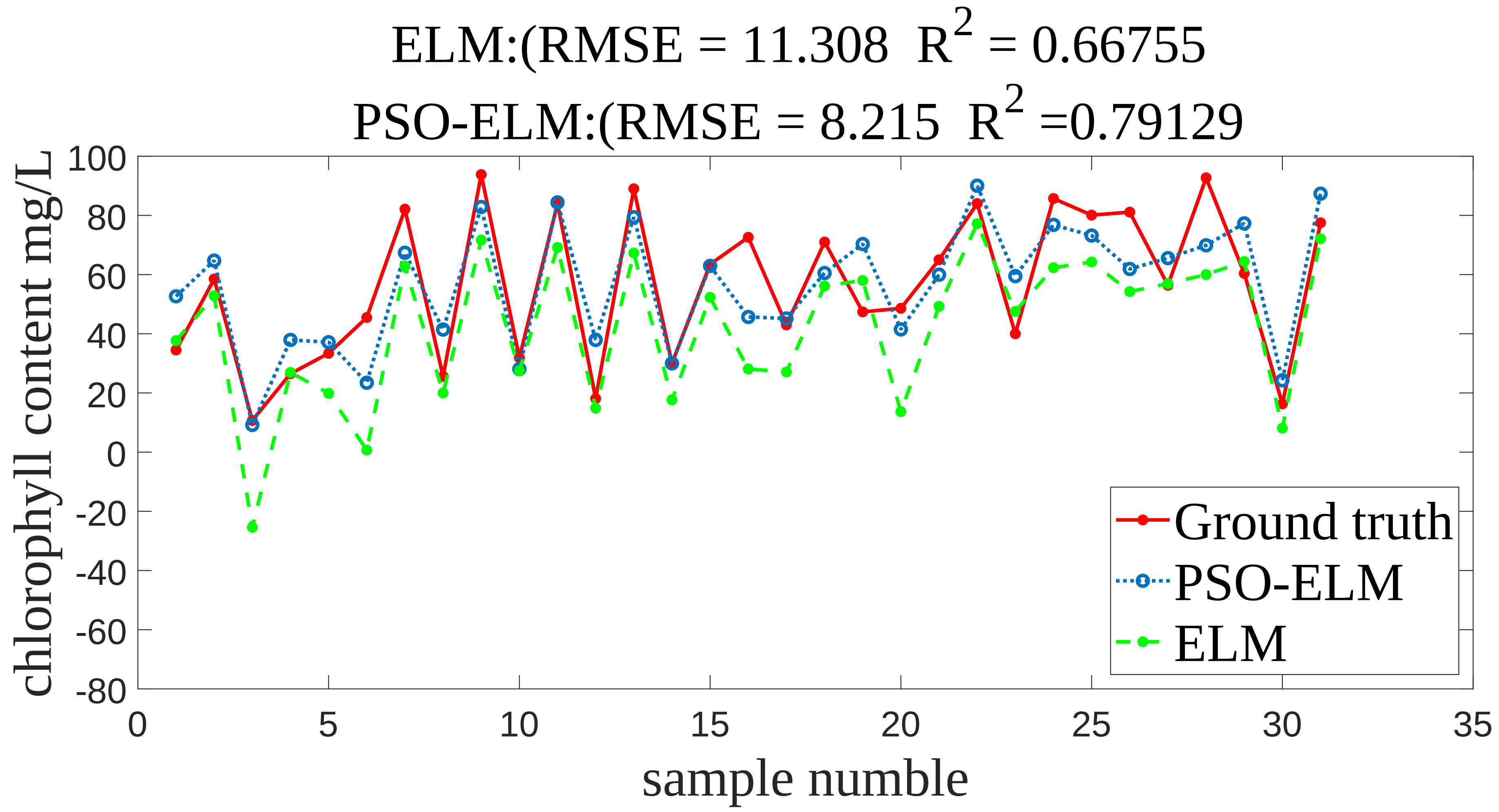

Supplement: Attachment — Submitted filename: PONE-D-20-07987_reviewer-.pdf [file pone.0238530.s001.pdf]
