# Supplementary material for: Inversion modeling of japonica rice canopy chlorophyll content with UAV hyperspectral remote sensing
Source: PLoS One. 2020 Sep 11;15(9):e0238530. doi: 10.1371/journal.pone.0238530 (PMC7485794; doi:10.1371/journal.pone.0238530)
Supplement: Attachment — Submitted filename: response.pdf [file pone.0238530.s002.pdf]

Response to Reviewers: PONE-D-20-07987

Title: *Inversion Modeling of Japonica Rice Canopy Chlorophyll Content with UAV Hyperspectral Remote Sensing*
Authors: *Yingli Cao, Kailun Jiang, Jingxian Wu, Fenghua Yu, Wen Du, Tongyu Xu*
Journal: *PLOS ONE*

The authors thank the editor and the reviewers for their careful reviews and high quality review comments, which have significantly helped us revise and improve the quality of the paper. The comments are very constructive and extremely helpful during the revision process. All of the reviewers' comments have been addressed in the revised version.

Below are the detailed point-to-point responses to their comments, and the corresponding modifications based on their suggestions.

1 Response to Comments of Reviewer 1

Thanks for your careful review and valuable comments for the improvement of this paper. The authors appreciate your detailed comments and valuable suggestions on the technical contents. Your technical concerns and suggestions are addressed as follows.

In this work, the possible use of UAV-HSI to predict the Chlorophyll content was evaluated. However, several questions should be explained or improved, including the writing expression, they are listed below:

1. **Why use the HSI combined with UVA to detect the chlorophyll content? The introduction of significance of this work should be improved, especially in Section Introduction and Section Discussion.**

   Response:

   Thanks for your question regarding the combination of HSI and UAV, and your suggestion on the description of the significance of the work.

   First, the combination of HSI and UAV can provide rapid, accurate, and non-destructive access for rice growth information. Conventional optical sensors cover only the visible light spectrum. On the other hand, the HSI sensors cover both the visible light spectrum and part of the infrared spectrum. Thus the HSI sensor has a much broader spectrum than optical sensors. Based on the results obtained in this paper, the hyperspectral information obtained by the HSI sensor contain sensitive features that are directly related to chlorophyll

contents, yet those information are not available in the visible light spectrum. Therefore, employing HSI can provide the necessary information to build a more accurate chlorophyll content inversion model.

Second, the UAV platform enables rapid and non-destructive data collection from the rice field. The UAV platform enables timely understanding of the rice growth status, which is essential for rice fertilization planning. Traditional methods of measuring crop growth indicators (chlorophyll content, leaf area index, biomass, etc.) are mainly manual measurements with indoor Chemical analysis, which is time-consuming and labor intensive. The manual approach is often poorly timed, and it seriously limits the timeliness and effectiveness of crop growth monitoring. The combination of HSI and UAV, on the other hand, can provide rapid and accurate assessment of the crop growth status.

Therefore, the development of hyperspectral monitoring system on UAV platforms can significantly improve the efficiency and accuracy of growth status monitoring and precision management of rice field. Such a system can facilitate the development of precision agriculture system, increase crop yields, and promote the socio-economic development of the society.

The above points have been elaborated in the Introduction section of the revised paper.

2. **The data processing without any black-white correction is not enough rigorous. Raw data from HSI contains tremendous amount of information including samples and noises. It's very important to eliminate the interference of the natural lighting (or weather conditions) in this work. So, in my opinion, the system with BW correction can promote the applicability of UAV-HSI.**

   Response:

   Thanks for your suggestion with black-white correction. We agree that BW correction is important for HSI image processing. Actually all results in the original manuscript were obtained after BW correction. Specifically, hyperspectral reflectance calibration was performed to achieve BW correction. The hypersectral reflectance calibration is performed as follows:

$$\rho_t = \frac{DN_t - DN_1}{DN_2 - DN_1}(\rho_2 - \rho_1) + \rho_1$$

   where ρ_t and DN_t are, respectively, the reflectivity and DN values of the ground objects to be converted, DN_1 and DN_2 are the DN values of the calibration blanket, and ρ_1 and ρ_2 are the reflectivity of the calibration blanket.

   The above information is included in Section II-B of the revised paper.

3. **From the figures 4 to 6, the spectral reflectance in the range of 400 and 450**

nm was lower than 0.1, which means low SNR (Signal to noise ratio) in this wavelengths, why did the authors still use the data to do further analysis?

Thanks for your question regarding the signal-to-noise ratio (SNR) between the band 400 to 450 nm. The results shown in Fig. 4 are obtained after noise reduction by using Savitzky-Golay convolution smoothing. Even though the reflectance is lower than 0.1 in that range, the data still contains valid information about the chlorophyll content. Motivated by your comments, we performed additional analysis regarding the SNR at different spectrum. The SNR of each band is obtained by calculating the ratio between the total power of the signal and the variance of the signal in the range between 400 to 800 nm. The SNR of all bands is between 6.0 dB to 15.9 dB, which is sufficient to extract the characteristic information of the response.

The above point has been clarified in Section III-A of the revised paper.

4. **For the selection of key spectral wavelengths, normally one detect the OD values at 663 nm and 645 nm with spectrophotometer, and then calculate the values with equations to describe the contents of chlorophyll contents, and thus these two wavelengths should be selected as key wavelengths.**

   Response:

   Thanks for your question and suggestion regarding key spectral wavelengths.

   The 663 nm and 645 nm wavelengths are primarily used by conventional chlorophyll measurement with Spectrophotometer, which is quite different from the approach proposed in this paper. The principle of spectrophotometer measurement is to measure the absorption intensity of chlorophyll solution at different wavelengths, and the strength of chlorophyll content can then be deduced from the absorption intensity at different wavelengths. For spectroscopic measurement, most of the essential information is expressed at the characteristic wavelengths of 663 nm and 645 nm.

   However, the data collected in this study is the reflectance information of leaves, which are solids instead of solutions. The leaf reflectance measured in this study is fundamentally different from the absorption intensity of the ethanol solutions of grounded rice leaves. Thus the reflectance characteristic wavelengths identified in this study are different from those absorption intensity wavelengths used in spectrophotometer measurement.

   The characteristic wavelengths identified in this study are obtained by analyzing the statistical correlation between leaf reflectance and chlorophyll contents, and the bands that shows stronger correlations are chosen during the construction of the inversion model. The experiment results show that the characteristic wavelengths selected in this work can be used to obtain accurate estimation of chlorophyll contents in the leaves without the need of ethanol solutions.

The above explanations have been added in Section II-C of the revised manuscript.

5. **Large area of the paddy field were selected as regions of interest, why only four japonica rice plants in test plots were used for chemical experiments?**

   Response:

   Thanks for your question about the possible confusion in the number of rice samples used for the chemical experiments. There is possibly a misunderstanding about the sample numbers. During chemical experiment, a total of 196 rice plant samples were collected and analyzed. Each sample is a four-pole rice sample, and there are a total of 196 of them. The four rice cavities selected for the same sample for this study were based on the similarity of rice growth in the rice experimental area.

   The above explanations have been added in Section II-C of the revised manuscript.

6. **The sample division for calculated and predicted datasets should be explained and provided in the manuscript.**

   Response: Thanks for your question regarding sample division. During the study, the entire dataset of 196 samples are randomly divided into two sets for training and testing, respectively. The training set contains 84% (165 samples) of the total data , and the remaining 16% (31 samples) is in the testing set. The data division was also summarized in Table I of the revised paper.

   This is clarified in Section II-C and Table I of the revised paper.

7. **Method for UAV-HSI data acquisition is unclear.**

   Response:

   Thanks for your suggestion to improve the method description of UAV-HSI data acquisition. Following your suggestion, we have added more detailed description of the UAV-HSI data acquisition process in Section II of the revised paper. They are as follows.

   The UAV hyperspectral platform adopts the M600 PRO six-rotor UAV from Shenzhen Dajiang Innovation Co., Ltd. (Shenzhen, Guangdong, China). The hyperspectral images were collected by using the GaiaSky-mini built-in push-broom airborne hyperspectral imaging system from Sichuan Shuangli Hepu Company (Sichuan, China). The hyperspectral band range is 400 to 1000 nm, with a 3 nm resolution. The total number of effective bands is 253.

   The Gaiasky-mini airborne hyperspectral imaging system is used to obtain hyperspectral remote sensing images of the rice canopy. The hyperspectral imaging sensor has a built-in push-sweep imaging function, thus the hyperspectral image was obtained by keeping the drone hovering steadily over the rice fields during the data acquisition process. During data acquisition, the drone is hovered with an altitude of 150 m and stationary to the ground.

and the corresponding coverage area of one hyperspectral scene image is 1000 m^2. The acquisition time of a scene image is 15 seconds. All hyperspectral images used in this work are obtained during the rice fertility period.

The above information is included in Section II-B of the revised paper.

8. **Unit for RMSE should be provided.**

   Response:

   Thanks for your suggestion with the unit for RMSE. The unit of RMSE in this study is mg/L.

9. **Why PSO-ELM was employed?**

   Response:

   Thanks for your question regarding the the employment of the PSO-ELM algorithm.

   Extreme learning machine (ELM) is a feedforward neural network with a single or multiple hidden layers. Unlike conventional neural network with back propagation (BP), the parameters of the nodes in the hidden layers of ELM are randomly assigned and never tuned. One main advantage of ELM is that it can learn much faster compared to conventional neural network with BP. However, due to the randomness of the parameters of the hidden layer nodes, a large number of hidden layer nodes are usually needed to achieve the desired accuracy in many practical applications. In addition, the conventional ELM architecture sometimes does not have good generalization capability.

   To address the above issues, we propose a particle swarm limiting learning machine algorithm, which uses a combination of particle swarm optimization (PSO) and limiting learning machine networks. Specifically, the PSO algorithm is used to optimize the weights of the input layer and the deviations of the hidden layer. Specifically, the PSO algorithm is used to optimize the weights of the input layer and the deviations of the hidden layer. In this case, the number of hidden layer nodes is learned automatically from the data, such that a good optimization performance can be achieved with a relatively small number of hidden layer nodes with low complexity.

   The above explanations have been added in the Section I of the revised manuscript.

10. **It would be better if authors can discuss the actual values and practical challenges of the UVA-HSI in modeling of chlorophyll content prediction.**

    Response:

    Thanks for your suggestion about the actual values and practical challenges of UAV-HSI for chlorophyll content prediction.

One of the main actual values of the proposed UAV-HSI platform is that it enables rapid, accurate, and non-destructive assessment of rice growth information. The method developed in this paper enables us to obtain timely understanding of the rice growth status, which is essential for rice fertilization planning.

One challenge faced by the proposed method is that the chlorophyll content of rice leaves varies with changes in rice varieties, fertility period, growing environment and other factors. The results obtained in this work are applicable to the crop stage of northeastern rice. New model parameters will need to be obtained for other rice strains.

Another challenge is that the airbone hyperspectral imaging system is sensitive to weather and lighting conditions. This challenge can be addressed by performing data acquisition at around noon on sunny days to ensure the weather condition and illumination conditions are similar in different data batches.

The above discussions have been added in the Section III-C of the revised paper.

2 Response to Comments of Reviewer 2

Thanks for your careful review and valuable comments for the improvement of this paper. The authors appreciate your detailed comments and valuable suggestions on the technical contents. Your technical concerns and suggestions are addressed as follows.

1. **Page 1: Introduction may need to be reorganized.**

   Response:

   Thanks for your suggestion regarding the organization of the introduction. Following your suggestion, we have reorganized the introduction, and added new contents regarding the motivation and significance of the proposed method. In addition, we also provided detailed justifications of the architecture of the data acquisition system with unmanned aerial vehicle (UAV) and hyperspectral imaging (HSI), and the motivation and justification for the development of the particle swarm optimization (PSO) and extreme learning machine (ELM) algorithm. All changes in the introduction are highlighted in blue in the revised paper.

2. **Page 1: You may need to summarize the differences and shortcomings of these studies, and elicit the significance of your research.**

   Response:

   Thanks for your suggestion regarding the differences and shortcomings of the results in the literature. There are two main differences between these existing studies and the proposed research.

   First, the hyperspectral data of most of the existing works were collected on the ground or at low altitude. Therefore the coverage area of the measurement is very small. On the other hand, the proposed UAV platform can operate at a higher altitude, which enables efficient large scale data collection. Specifically, the proposed UAV-HSI platform operates at an altitude of 150 m, and can obtain the hyperspectral image of an 100 m^2 area in 15 seconds.

   Second, many existing methods in the literature were developed by using traditional regression methods, where many of the model parameters were initialized in a heuristic manner based on past experience. The proposed approach using a data-drive approach, such that all model parameters, including the characteristic bands and weights of the PSO-ELM algorithm, are obtained through the collected data. The data-driven approach can remove human bias, and obtain a more accurate prediction results.

   The above statement has been added in the Introduction of the revised manuscript.

3. **Page 2: The introduction does not introduce the reference of the method used in your research (PSO-ELM)**

   Response:

   Thanks for your suggestion with the reference of the methods. Following your suggestion, we have added the reference for PSO [15], ELM [16], and PSO-ELM [19].

4. **Page 2: reference?**

   Response:

   Thank you for your question. We have added the references in the revised paper as follows:

   In addition, the conventional ELM architecture sometimes does not have good generalization capability [17] [18]. To address the above issues, we propose to employ a particle swarm limiting learning machine algorithm that employs a combination of PSO and ELM [19]. Specifically, the PSO algorithm is used to optimize the weights of the input layer and the deviations of the hidden layer. In this case, the number of hidden layer nodes is learned automatically from the data, such that a good optimization performance can be achieved with a relatively small number of hidden layer nodes with low complexity. It has been demonstrated in this paper that the PSO-ELM algorithm outperforms conventional ELM algorithm in terms of the number of hidden layer nodes and network generalizations.

5. **just flying height? please add some detailed information such as flying speed, flying overlapping and so on**

   Response:

   Thanks for your suggestion regarding the UAV flight parameters. Following your suggestion, we have added more description about the UAV flying parameters and data acquisition methods. They are added in Section II of the revised paper as follows.

   The UAV hyperspectral platform adopts the M600 PRO six-rotor UAV from Shenzhen Dajiang Innovation Co., Ltd. (Shenzhen, Guangdong, China). The hyperspectral images were collected by using the GaiaSky-mini built-in push-broom airborne hyperspectral imaging system from Sichuan Shuangli Hepu Company (Sichuan, China). The hyperspectral band range is 400 to 1000 nm, with a 3 nm resolution. The total number of effective bands is 253.

   The Gaiasky-mini airborne hyperspectral imaging system is used to obtain hyperspectral remote sensing images of the rice canopy. The hyperspectral imaging sensor has a built-in push-sweep imaging function, thus the hyperspectral image was obtained by keeping the drone hovering steadily over the rice fields during the data acquisition process. During data acquisition, the drone is hovered with an altitude of 150 m and stationary to the ground. and the corresponding coverage area of one hyperspectral scence image is 1000 m^2. The

acquisition time of a scene image is 15 seconds. All hyperspectral images used in this work are obtained during the rice fertility period.

6. **Page 2: The data processing process needs to add some specific operations and parameter settings.**

   Response:

   Thanks for your question regarding the data processing process. Following your suggestion, we have added more details about data processing procedure in the revised manuscript in Section II-B, and details are given as follows.

   The collected hyperspectral data were processed and extracted with the ENVI5.3+IDL software tool. During processing, the spectral angle mapper (SAM) method was first used to remove impacts from interfering objects, then the hyperspectral image was generated by calculating the average spectrum of each area of interest. Before data analysis, hyperspectral reflectance calibration was performed to achieve black-white correction in the HSI image. The hypersectral reflectance calibration is performed as follows:

   $$\rho_t = \frac{DN_t - DN_1}{DN_2 - DN_1}(\rho_2 - \rho_1) + \rho_1$$

   where ρ_t and DN_t are, respectively, the reflectivity and DN values of the ground objects to be converted, DN_1 and DN_2 are the DN values of the calibration blanket, and ρ_1 and ρ_2 are the reflectivity of the calibration blanket.

7. **hyperspectral image was generated? or hyperspectral reflectance**

   Response:

   Thanks for your question. The pixels in the hyperspectral image are obtained by using hyperspectral reflectance data, that is, each pixel in the image contains the hyperspectral reflectance information at the corresponding location. The above point has been clarified in Section I Introduction of the revised paper.

8. **how to measure the ground true data. What is the name of the instrument? What are the specific parameters and operating instructions of the instrument?**

   Response:

   Thanks for your question about the measurement and collection of ground true data. The instrument used in ground data measurement is Hengping 754 UV-visible spectralphotometer. It covers the wavelength range from 190 nm to 1000 nm, with a step size of 0.1 nm. Details of the ground true data measurement process are given in the revised paper as follows.

   After the japonica rice samples were returned to the laboratory, fully-expanded leaves of the japonica rice samples were selected and cut into small pieces. Pieces from the same leaf are

mixed together, and 0.4 g of the leaf mixture was placed in 200 mL of an extraction solution, which contains acetone, ethanol, and distilled water at a ratio of 9:9:2. The solutions with leaf mixture are left to stand in the shady area of a laboratry with temperature around 20°C. Once the leaf sample appeared to be completely white, colorimetry was then performed with a Hengping 754 UV-visible spectralphotometer. The spectralphotometer covers the wavelength range from 190 nm to 1000 nm, with a step size of 0.1 nm. The spectrophotometer is to measure the optical density (OD) at 663 nm and 645 nm, respectively. The principle of spectrophotometer measurement is to measure the absorption intensity of chlorophyll solution at different wavelengths, and the strength of chlorophyll content can then be deduced from the absorption intensity at different wavelengths. For spectroscopic measurement, most of the essential information is expressed at the characteristic wavelengths of 663 nm and 645 nm. It should be noted that the proposed UAV-HSI system is measuring the reflectance information of leaves in solid form, thus the reflectance characteristic wavelengths used by the UAV-HSI platform will be different from 663 nm and 645 nm.

9. **the accuracy of the instrument?**

   Response: The manufacture of the instrument is XAG, and the model is RTK. The precision of the GPS device is on the order of a few centimeters. The above information has been added in the revised manuscript as follows.

   During the collection of the ground data, a hand-held differential GPS device (manufacture: XAG, model: RTK, precision: centimeters) was used to measure the geographical coordinates of the sampling points.

10. **how to correlate? can you add some detailed information?**

    Response:

    Thanks for your question regarding with the correlation between the ground data and HSI data. The ground data and HSI data are correlated by using GPS information. During the collection of the ground data, a hand-held differential GPS device (manufacture: XAG, model: RTK, precision: centimeters) was used to measure the geographical coordinates of the sampling points. The HSI data also contains GPS information of each pixel. The ground data of a given sample are then mapped to a pixel in the HSI data with the same GPS coordinate.

    The above procedure has been clarified in Section II-C the revised paper.

11. **This part only has results, lack of analysis**

    Response:

    Thanks for your comments. Following your suggestion, we have combined Section III Results and Analysis, and Section IV Discussion, into a new section: Section III Results and

Discussion. In the new discussion subsection, we added new discussions about the significances and challenges of the proposed UAV-HSI platform with the PSO-ELM algorithm. All changes are highlighted in the revised paper.

12. **The discussion part is like a conclusion**

    Response:

    Thanks for your comments regarding the discussion section. In the revised paper, we have combined Section III Results and Analysis, and Section IV Discussion, into a new section: Section III Results and Discussion. In the new discussion subsection, we added new discussions about the significances and challenges of the proposed UAV-HSI platform with the PSO-ELM algorithm. All changes are highlighted in the revised paper.
