# Supplementary material for: Inversion modeling of japonica rice canopy chlorophyll content with UAV hyperspectral remote sensing
Source: PLoS One. 2020 Sep 11;15(9):e0238530. doi: 10.1371/journal.pone.0238530 (PMC7485794; doi:10.1371/journal.pone.0238530)
Supplement: Attachment — Submitted filename: response.pdf [file pone.0238530.s003.pdf]

Response to Reviewers: PONE-D-20-07987

Title: *Inversion Modeling of Japonica Rice Canopy Chlorophyll Content with UAV Hyperspectral Remote Sensing*
Authors: *Yingli Cao, Kailun Jiang, Jingxian Wu, Fenghua Yu, Wen Du, Tongyu Xu*
Journal: *PLOS ONE*

Response to Comments of Reviewer

Thanks for your careful review and valuable comments for the improvement of this paper. The authors appreciate your detailed comments and valuable suggestions on the technical contents. Your technical concerns and suggestions are addressed as follows.

1. **Is it a mistake saying the hyperspectral band range is 400 to 100 nm and with a 3 nm resolution? Why the number of effective bands is 253? Please have a check and make necessary changes (100-1000 nm)**

   Response:

   Thanks for your question. We have clarified the number of effective bands in Section II-B of the revised paper as follows:

   The frequency range of the data acquired by the hyperspectral imaging sensor is 400 to 1000 nm. The data is preprocessed by using spectral difference calculations with a hyperspectral resampling interval set to 2.35 nm, which results in 255 bands over the spectral range between 400 to 1000 nm. The two bands on the upper and lower boundaries of the spectrum are removed, which leads to 253 effective bands.

2. **Although the authors added some references (33-38), it would be better to discuss further or compare your results with the published data**

   Response:

   Thanks for your suggestion about further comparison with the published data in the literature. Following your suggestion, we compared the characteristics bands obtained in this paper and those published in the literature. The five characteristic bands extracted in this study are 410 nm, 481 nm, 533 nm, 702 nm, 798 nm. The two bands in 702 nm and 798 nm are are consistent with the 705 nm and 750 nm range used for chlorophyll content retrieval index in [33]. The characteristic bands of 410 nm, 481 nm, and 533 nm in this paper are similar to those bands used in the study of the rice jointing stage in the literature [34]. Comprehensive analysis shows that there are overlaps and differences in the spectral regions of

the characteristic bands among different studies. The differences in characteristic bands are mainly caused by differences in strain varieties, growth periods, environmental conditions, and data processing methods.

The above point is clarified in Section IV of the revised paper.
